# The case for character displacement in plants

**DOI:** 10.1002/ece3.978

**Published:** 2014-02-14

**Authors:** Carolyn M Beans

**Affiliations:** Department of Biology, University of VirginiaCharlottesville, Virginia

**Keywords:** Ecological character displacement, niche differentiation, plant–plant interactions, reproductive character displacement

## Abstract

The evidence for character displacement as a widespread response to competition is now building. This progress is largely the result of the establishment of rigorous criteria for demonstrating character displacement in the animal literature. There are, however, relatively few well-supported examples of character displacement in plants. This review explores the potential for character displacement in plants by addressing the following questions: (1) Why aren't examples of character displacement in plants more common? (2) What are the requirements for character displacement to occur and how do plant populations meet those requirements? (3) What are the criteria for testing the pattern and process of character displacement and what methods can and have been used to address these criteria in the plant literature? (4) What are some additional approaches for studying character displacement in plants? While more research is needed, the few plant systems in which character displacement hypotheses have been rigorously tested suggest that character displacement may play a role in shaping plant communities. Plants are especially amenable to character displacement studies because of the experimental ease with which they can be used in common gardens, selection analyses, and breeding designs. A deeper investigation of character displacement in plants is critical for a more complete understanding of the ecological and evolutionary processes that permit the coexistence of plant species.

## Introduction

Patterns of altered morphology in sympatric versus allopatric populations have long been described in the plant literature. *Pachycereus pringlei* grows taller where its range overlaps with other cacti species (Cody 1984), *Solanum grayi* blooms smaller when in contact with *Solanum lumholtzianum* (Whalen 1978), and *Arenaria uniflora* shifts from outcrossing to selfing where intermixed with *Arenaria glabra* (Fishman and Wyatt 1999). Character displacement, first defined in the animal literature, is frequently used to explain these patterns. Character displacement is the process whereby competing species respond to selection to increase their mean difference in a trait associated with resource use or reproduction (Brown and Wilson 1956; Mayr 1970; Pfennig and Pfennig 2009). The end result is a divergence in phenotypes, which reduces the overlapping resource needs or reproductive interactions of the species and permits their coexistence. This process of character displacement results in a pattern of competitors exhibiting greater trait divergence in regions of sympatry than in regions of allopatry (Brown and Wilson 1956).

Character displacement was first described in animals by Brown and Wilson (1956). Following this publication, both plant and animal biologists began documenting cases of character divergence in sympatric species and attributing these patterns to character displacement. Many of these early studies attracted controversy because they relied solely on patterns that could otherwise be explained by any number of factors, such as variation in resource availability between sympatric and allopatric populations, ecological sorting, or even chance (Mayr 1963; Grant 1975; Arthur 1982; den Boer 1986).

Despite the initial controversy, the evidence for character displacement as a widespread response to harmful competitive or reproductive interactions is now building (Losos 2000; Pfennig and Pfennig 2009). This progress is largely the result of the establishment of rigorous criteria for demonstrating character displacement (Grant 1994). These criteria include ruling out alternative hypotheses such as chance or ecological sorting, as well as establishing that the character divergence is driven by interspecific competitive or reproductive interactions (Taper and Case 1991).

Much progress has been made since the establishment of these criteria, but the support for character displacement still rests primarily on a limited number of animal species (i.e., *Plethodon* salamanders (Adams et al. 2007), sticklebacks (Pritchard and Schluter 2001), finches (Grant and Grant 2006), *Anolis* lizards (Losos and Spiller 1999), Mexican spadefoot toads (Martin and Pfennig 2011)). Although theoretical and experimental frameworks for studying character displacement in plants were developed decades ago (Levin 1970; Fowler and Antonovics 1981), there are still relatively few well-supported examples of plant character displacement (Schluter 2000a; Dayan and Simberloff 2005).

The goal of this review was to inspire a greater focus on this field by pointing to the lack of character displacement studies in plants and by offering tools – both new and old – for rigorously testing the character displacement hypotheses in plant systems. I will explore our current understanding of character displacement in plants by addressing the following questions: (1) Why aren't examples of character displacement in plants more common? (2) Under what circumstances is character displacement likely to occur in plants? (3) What are the criteria for testing the pattern and process of character displacement and what methods can and have been used to address these criteria in the plant literature? (4) What are some additional approaches for studying character displacement in plants? A deeper investigation into character displacement in plants is critical for a more complete understanding of ecological and evolutionary forces that shape plant communities.

## Background

There are two forms of character displacement – ecological and reproductive. I define ecological character displacement here as the evolution of morphological, behavioral, physiological, or developmental trait divergence in one or more sympatric species in response to interspecific competition for limited resources (Brown and Wilson 1956; Hansen et al. 2000). I define reproductive character displacement as the evolution of divergence in any of these same traits in one or more sympatric species in response to competition for the resource of pollinators, or in response to the costs associated with sharing pollinators with another species (Pfennig and Pfennig 2009). These costs may include the loss of pollen to other species, the clogging of stigmas with heterospecific pollen, or the potential for unfit hybrid seed.

Reproductive character displacement may occur between distantly or closely related species. Distantly related species may evolve in response to competition for pollinators or the fitness costs associated with heterospecific pollen transfer. Closely related species capable of hybridization may undergo reinforcement, the evolution of traits that minimize costly mating or hybridization between recently diverged species (Hopkins 2013), which is defined here as one form of reproductive character displacement (Pfennig and Pfennig 2009). While intraspecific plant competition may also lead to character displacement and subsequent speciation, within-species interactions are beyond the scope of this review.

The line between reproductive and ecological character displacement is sometimes unclear. For example, a trait such as the timing of seed germination may impact the competitive ability of the seedling (ecological character displacement) as well as the flowering period (reproductive character displacement; Armbruster 1986; Pfennig and Pfennig 2009).

Character displacement was first defined as a pattern of species trait divergence in regions of sympatry (Brown and Wilson 1956). Because this pattern could result from processes other than selection to reduce competitive or reproductive interactions, character displacement is now more commonly defined as the evolutionary process itself, rather than the resulting pattern (Pfennig and Pfennig 2009). This process of character displacement typically results in one of two patterns. The first is a shift in a trait mean where a species range overlaps with that of a competitor. The second is a pattern of overdispersion of trait means within an assemblage of ecologically similar species (Schluter 2000a).

Biologists have long sought to understand the relative importance of ecological, evolutionary, and stochastic forces in shaping plant communities (Cowles 1899; Clements 1916; Gleason 1926). The study of character displacement in plants has the potential to reveal whether competition-driven plant evolution plays an important role in this process. Both ecological and reproductive character displacements may promote the coexistence of species by enhancing niche differences and therefore reducing the magnitude of harmful interspecific interactions relative to intraspecific interactions (Hochkirch et al. 2007; Pfennig and Pfennig 2009). Ecological character displacement promotes species coexistence by reducing the competition for resources that might otherwise lead to competitive exclusion. Reproductive character displacement promotes species coexistence by reducing the competition for pollinators or costly interspecific reproductive interactions that might otherwise lead to low reproductive output and population decline described as “reproductive exclusion” (Pfennig and Pfennig 2009). The potential for a population to undergo character displacement therefore may mean the difference between survival in a new niche and local extinction. To date, however, we do not have enough examples of character displacement in plants to assess its relative role in structuring plant communities.

## Why aren't There More Examples of Character Displacement in Plants?

Despite its potential relevance to our understanding of basic ecological and evolutionary processes, earlier reviews have uncovered relatively few studies of character displacement in plants (Levin 1970; Schluter 2000a; Dayan and Simberloff 2005). I surveyed the character displacement literature to assess whether there are still relatively few studies in plants. In June of 2012, I conducted a Google Scholar search for publications that contained the term character displacement in the title. The search returned 323 results. I updated this search in December of 2013. From these results, I selected peer-reviewed studies of character displacement involving two or more species where new data were presented. I only included studies where the explicit goal was to test for character displacement. For example, there are many studies that investigate resource partitioning among competitors. I did not select these studies unless the authors tested whether resource partitioning was achieved through evolved trait differences rather than some other ecological interaction such as plastic responses to competition or ecological sorting.

**Figure 1:**
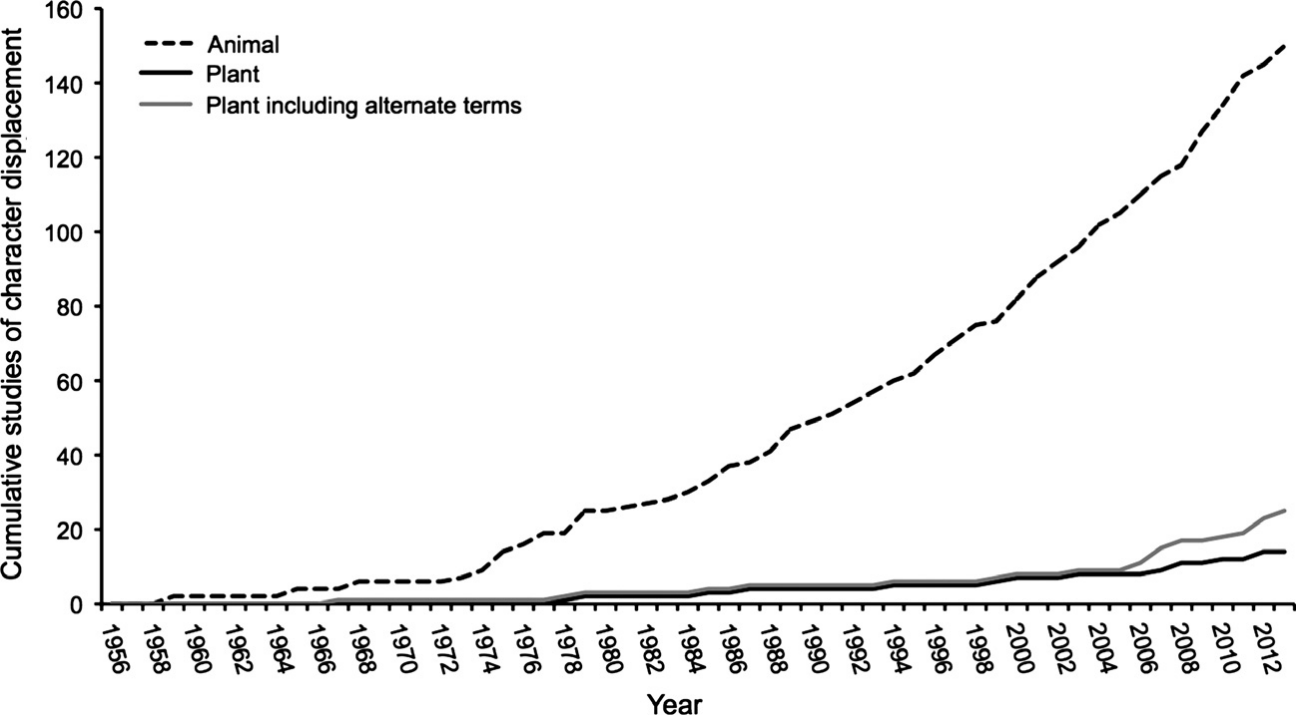
Cumulative studies of character displacement published from 1956 to December of 2013. The *Animal* and *Plant* categories include all studies that were published in each of these groups with the term “character displacement” in the title. The *Plant including alternate terms* category includes all studies testing for character displacement that used the term “character displacement” and/or alternate terms with similar meaning in the title.

The results of this survey show that there are still few published studies of character displacement in plants (Fig. 1, Table S1). Since 1956 when the term was coined, there have been 150 animal studies and only 14 plant studies for which character displacement was an important enough focus of the research to be included in the title. While my literature search was not exhaustive, as studies of character displacement exist that do not include the term in the title, these results strongly indicate an overall trend toward more character displacement research in the animal versus plant literature. Why are there so few examples of character displacement in plants?

### Language

The difference between the prevalence of character displacement studies in the animal versus plant literature may be an issue of language. Perhaps the plant literature describes the process of character displacement using different terms. To test this hypothesis, I compiled a list of alternate terms that may be used instead of character displacement to describe the same process. For ecological character displacement, I expected that the plant literature might also refer to this process as selection for evolution of niche partitioning or niche differentiation. For reproductive character displacement, I expected that the plant literature might also refer to this process as reinforcement, the Wallace effect, or natural selection for reproductive isolation. In June of 2012, I conducted an additional Google Scholar search for plant studies that included the alternate terms as follows: *plant AND selection AND intitle:* “*niche partitioning” OR intitle:* “*niche differentiation” OR intitle:* “*reproductive isolation” OR intitle:* “*reinforcement” OR intitle:* “*Wallace effect.”* This search returned over 1000 results. I updated this search in December of 2013. Of these results, I selected only plant studies where the authors tested for trait divergence in sympatry by investigating competition-driven selection or adaptation for niche differentiation, niche partitioning, reproductive isolation, reinforcement, or the Wallace effect. There were many studies that suggest reproductive isolation as a by-product of natural selection in allopatry, but these studies were not included because they do not test whether reproductive isolation was itself under selection in sympatry. I was left with 11 papers that could be interpreted as studies of character displacement, only five of which used the term character displacement in the body of the manuscript (Table S2). Nine papers used the term reproductive isolation in the title and two used the term reinforcement. The other search terms did not return relevant results. After adding these 11 papers to the original search, there were a total of 25 plant studies of character displacement (Fig. 1). Twenty of these studies suggested that character displacement was a possible explanation for the observed pattern. Still, while this additional search nearly doubled the total number of plant studies, there remain far fewer studies of character displacement in plants than in animals. Differences in language therefore do not explain the lack of plant studies.

### Theory

As noted in a recent review of plant interactions and evolution, the prevalence of theories that downplay the role of plant interactions in structuring plant communities may deter biologists from looking for an evolutionary response to these interactions (Thorpe et al. 2011). For example, the individualistic theory of plant distribution argues that plants are distributed according to their tolerances for different environmental conditions (Gleason 1926). Accordingly, a community of plant species is simply a grouping of species that share an affinity for the environmental conditions at a particular location. A species' place in this community is entirely independent of all other species in the community. Plant interactions therefore are unimportant, so there is no reason to predict they would drive evolutionary change. The neutral theory of plant community ecology also suggests that plant interactions are not critical to community assembly (Hubbell 2005). This theory argues that plant communities are constructed according to the processes of random speciation, random dispersal, and ecological drift. The random nature of plant community assembly under this theory ignores the competitive advantages and disadvantages of individual species. With all species treated as equal, there is no place for the competition that would otherwise drive an evolutionary response.

If these theories are correct, then character displacement may truly be a rare occurrence in plant communities. The lack of published studies of character displacement may reflect a bias toward only publishing positive results rather than a bias toward only studying character displacement in animals. If these theories are incorrect, however, as many studies suggest, their prevalence may be discouraging us from exploring a key component of species coexistence (Clements 1916; Odum 1971; Tilman 1981; Silvertown 2004; Wilsey et al. 2009; Thorpe et al. 2011).

### Detection bias

There may be fewer studies of character displacement in plants simply because fewer biologists study plants. It is also possible that character displacement in plants may be more difficult to detect. Shifts in nitrogen form uptake, rooting depth, or style length are not nearly as obvious as, for example, a shift from a carnivorous to an omnivorous morph of spadefoot toad (Martin and Pfennig 2011). Ecological character displacement may be especially difficult to detect. Although there are at least a couple of plant studies suggestive of ecological character displacement, all studies uncovered by the literature search except one were of reproductive character displacement (Cody 1991; Veech et al. 2000). Reproductive traits are among the showiest plant traits and therefore may attract the attention of researchers more so than ecological traits. Ecological traits susceptible to character displacement could be physiological or developmental rather than morphological and therefore much more subtle. Yet these character shifts may be equally prevalent. Biologists have long documented that the intensity of competition is lower for plant populations with a history of coexistence with a competitor than for with those that are naïve to the competitor. (Turkington 1989; Shaw et al. 1995; Mealor and Hild 2007). But because the specific trait shifts responsible for the decreased intensity of competition are often not identified, these studies have largely remained separate from the character displacement literature.

## Under What Circumstances is Character Displacement Likely to Occur in Plants?

For character displacement to occur, a population must first meet the basic requirements for evolution in response to natural selection (Antonovics 1978). Then, character displacement is only likely to occur if the initial difference in trait means between the two competitors is intermediate (Schluter 2000b). If there is too little difference in trait means, there will be an initial slow response to selection, and competition may become severe enough that one species may drive the other to local extinction, whereas if the difference is too large, then selection will not be strong enough to encourage further divergence (Antonovics 1978; Taper and Case 1991; Schluter 2000b; Pfennig and Pfennig 2009). It seems likely that plant populations could meet the above requirements. There are, however, additional factors that encourage character displacement, which some suggest may not apply to plant communities (Connell 1978, 1980; Keddy 1989).

### Repeated contact

For character displacement to occur, competitors must have frequent contact with one another to maintain a constant force of selection (Pfennig and Pfennig 2009). This scenario is common in animal systems. For example, constant competition between similar species of sticklebacks has repeatedly resulted in character displacement in foraging depth and food choice (Pritchard and Schluter 2001). Connell argued that plant competition is unlikely to result in this same sort of niche differentiation because competition-driven evolution is dependent on the frequency with which two species come into contact and most plants are sympatric with a wide variety of competitors (Connell 1980). According to this argument, selection acting in many directions on multiple pairs of species would overwhelm selection driven by a single competitor.

There may, however, be exceptions that would allow for frequent contact between plant competitors. For example, in invaded plant communities, native species come into frequent contact with a single dominant invasive (Leger and Espeland 2010; Thorpe et al. 2011). Also, a plant population may be surrounded by a variety of species, but only compete with one for a specific resource. *Dalechampia* species only compete with congeners for pollination because only specialized bees are drawn to their unique resin-producing glands (Armbruster 1985, 1986). Finally, although likely less common, multiple ecologically similar competitor species may all exert the same directional selection on a focal species. Under these circumstances, character traits in the focal species can evolve in response to the community of competitors (Cody 1991).

### Available niche space

Some argue that character displacement does not occur in plants because all plant species depend on the same resources (sun, water, nutrients) and therefore cannot diverge in form or function to divide the available niche space (Connell 1978; Keddy 1989). Evidence now suggests, however, that this assumption may be incorrect. Neighboring plant species have been shown to segregate according to microscale differences in habitat, thereby dividing up the available resource pool by specializing in different forms of resources (Fowler and Antonovics 1981). For example, differences in life-history traits in sympatric *Acer* species were associated with a division of light resources in a Japanese deciduous forest (Tanaka et al. 2008). Additionally, species in plant communities ranging from European wet meadows to South African fynbos were shown to segregate according to fine-scale hydrological gradients (Araya et al. 2011). Plants can also divide niche space by preferentially taking up different forms of nutrients (Silvertown 2004; Miller et al. 2007). For example, the success of competitively superior plants in a diverse alpine dry meadow community was attributed to their ability to increase their uptake of nitrogen in the form of ammonium when competitors drew on the same resource pool (Ashton et al. 2010).

Finally, plants can divide niche space along multiple resource gradients at once (Tilman 1982; Vellend et al. 2000). The co-occurrence of five species of goldenrod (*Solidago*) was explained by the species' affinities for different combinations of soil acidity, clay content, and soil moisture, as well as by differences in life-history traits. For example, *Solidago altissima* and *Solidago gigantea* were most commonly associated with circumneutral soils, while the other goldenrod species preferred more acidic soils. Although *S. altissima* and *S. gigantea* shared a soil acidity niche, this niche space was further divided along a moisture gradient with *S. gigantea* associated with wetter soils (Abrahamson et al. 2005).

### Potential for phenotypic plasticity

Character displacement is especially common among animal species that display phenotypic plasticity (Rice and Pfennig 2007). Character displacement may occur more readily in plastic species because plasticity permits survival among competitors long enough for selection to narrow the reaction norm of each species in opposite directions, or potentially produce a more fixed sympatric phenotype (Rice and Pfennig 2007).

Plants frequently respond plastically to competitors (van Kleunen and Fischer 2001; Callaway et al. 2003; Fan et al. 2008; Burns and Strauss 2012). When competing for light, the stoloniferous plant, *Trifolium repens,* altered branching number and length, petiole elongation, leaf mass, and specific leaf area (SLA) differently in response to pairwise competition with competitors of varying growth forms (Bittebiere et al. 2012). Similarly, the coastal shrubs, *Haplopappus ericoides* and *H. venetus* var. *sedoides*, responded to competition for water with the invasive succulent, *Carpobrotus edulis*, by developing deeper rooting systems (D'Antonio and Mahall 1991). The plastic nature of these responses could increase the likelihood of character displacement in these species.

## What are the Criteria for Testing the Pattern and Process of Character Displacement and What Methods Can and have been Used to Address these Criteria in the Plant Literature?

Early evidence for character displacement in plants and animals rested primarily on correlational studies, which demonstrated the patterns of competitor species diverging in phenotype in regions of sympatry. This correlational evidence alone was not convincing, as any number of alternative hypotheses might also explain the observed patterns (Grant 1975; Arthur 1982; den Boer 1986). In response to the early criticism of character displacement studies, a set of criteria for demonstrating that a pattern is the likely result of character displacement was established in the animal literature. The criteria below were compiled by Schluter and McPhail (1992) and were then elaborated on by Taper and Case (1991) and Pfennig and Pfennig (2012).

1. The character displacement pattern was not formed by chance.
2. Difference in the trait of interest between sympatric and allopatric populations is genetically based.
3. Differences in character traits are the result of in situ evolution and not ecological sorting.
4. A shift in the trait of interest is associated with a shift in resource acquisition or reproductive interactions.
5. The strength of interspecific competition or reproductive interactions is positively correlated with the degree of phenotypic similarity between species.
6. Sympatric and allopatric sites have similar resource availability, which, in the case of reproductive character displacement, includes a similar diversity and abundance of pollinators.

The first three criteria rule out alternative hypotheses to in situ character divergence, and the last three address whether this character divergence is driven by interspecific interactions (Taper and Case 1991). While few studies meet all of these criteria, the growing number of studies in the animal literature that address four or more of them suggests that character displacement may be a widespread response to competition in animal communities (Schluter 2000a). In plants, the evidence is not yet as convincing. However, as described below, the studies that have attempted to address some of these criteria are strongly suggestive of character displacement.

Still, whether studying animals or plants, these criteria only require an exploration of existing patterns of character divergence. They do not investigate the process of character displacement itself. To prove character displacement is occurring, experiments must be designed to investigate the process of character displacement directly (Littlejohn and Loftus-Hills 1968; Losos 2000; Schluter 2000a; Stuart and Losos 2013). These experiments have rarely been attempted in either the animal or plant literature, but recent experimental studies in plants have found support for character displacement (Muchhala and Potts 2007; Hopkins et al. 2012).

### Addressing established criteria for explaining character displacement patterns

Below I illustrate the six criteria that must be addressed in order to demonstrate that an existing pattern of character divergence is the result of character displacement. For each criterion, I offer examples of plant studies that have satisfied it. To date, much of the evidence for character displacement in plants rests on the *Dalechampia*,*Burmeistera*, and *Phlox* systems (Table 1).

**Table 1:** Selected studies suggestive of character displacement in plants. Numbered columns mark whether studies were designed to address the criteria for testing the pattern of character displacement. Studies that experimentally test the process of character displacement often, as a by-product of the experiment, satisfy some or all of the criteria for explaining the existing pattern, but only studies designed to address the specific criteria are marked.

System	Studies	Trait(s) studied	Ecological (E) or Reproductive (R)	(1) Pattern is not formed by chance	(2) Trait differences are genetically based	(3) Trait differences result from in situ evolution	(4) Shift in trait is associated with shift in resource aquisition	(5) Similar phenotypes compete more strongly	(6) Sympatric and allopatric sites have same resources	Experimental test of CD process	Correlational evidence only
*Dalechampia* species	Armbruster (1985, 1986)	Resin gland area, gland-stigma distance, anther–stigma distance	R	X	X	X	X				
*Ipomopsis aggregata* and *Castilleja linariaefolia*	Caruso (2000)	Corolla length	R							X	
*Pachycereus pringlei* and other cacti species	Cody (1984)	Growth form	E								X
*Arenaria uniflora* and *Arenaria glabra*	Fishman and Wyatt (1999)	Mating system (selfing vs. outcrossing)	R				X			X	
*Phlox drummondii* and *Phlox cuspidata*	Levin (1985); Hopkins and Rausher (2011); Hopkins et al. (2012)	Corolla color	R		X		X	X		X	
*Burmeistera* species	Muchhala and Potts (2007); Muchhala (2008)	Exsertion length of anthers and stigma	R	X		X	X	X		X	
*Ipomea hederacea* and *Ipomea purpurea*	Smith and Rausher (2008)	Clustering of anthers around stigma	R							X	
*Pinus* species	Veech et al. (2000)	Seed mass	E	X							
*Solanum* section *Androceras*	Whalen (1978)	Floral size and phenology	R				X				

#### 1. Character displacement pattern not formed by chance

Models that are equally applicable to plant and animal studies are now available to help distinguish between character displacement and chance patterns. For example, when multiple sympatric and allopatric populations exist for a pair of species, the differences in mean phenotype between competing species in sympatry can be tested against a null model that generates the differences in phenotype between pairs of allopatric communities of the two species sampled at random (Losos 2000). When multiple assemblages with overdispersed trait means exist, the average degree of dispersion of trait means for multiple communities of sympatric species can be compared against that of a null model where populations of species are randomized across communities (Schluter 2000a; Muchhala and Potts 2007). These models have been successfully used in plant systems to reject the alternative hypothesis that patterns of character divergence are caused by chance (Armbruster et al. 1994; Stone et al. 1998; Veech et al. 2000; Muchhala and Potts 2007). Additionally, direct experimental tests of the process of character displacement, which will be discussed in the following section, can also satisfy this criterion Fishman and Wyatt (1999); Smith and Rausher (2008)).

#### 2. Difference in the trait is genetically based

A putative character displacement pattern may actually be caused by plastic responses to differences in environmental conditions or competitors between sites. To rule out this alternative hypothesis, the differences in phenotype between sites must be shown to have a genetic basis. The simplest method for testing for a genetic basis for trait differences between populations is a common garden experiment where individuals from one species taken from both sympatric and allopatric populations are grown in a common environment. If there is a genetic basis to the difference in trait means, then this same difference should be apparent when individuals from the two types of populations are grown together. It should be noted, however, that plasticity itself can be heritable and so can also potentially evolve in response to competition Pfennig and Pfennig (2012). To test whether plasticity itself has been displaced, common garden experiments can be used to test for differences in reaction norms between individuals from sympatric and allopatric populations growing with and without the competitor. Reciprocal transplants between sympatric and allopatric sites can also be used to test for evolved plasticity.

As the names of the methods imply, plants make especially suitable subjects for common garden designs and reciprocal transplants. When testing for a genetic basis for trait differences, Armbruster (1985) grew both live rootstock and seeds from multiple populations of *Dalechampia scandens* in a common greenhouse environment. In the source populations, *D. scandens* appeared to diverge in resin gland size, gland–stigma distance, and anther–stigma distance in response to various combinations of sympatric congeners. Measurements of these reproductive traits for individuals in the greenhouse matched the measurements of individuals from their source populations, greatly strengthening the case for character displacement in this system.

#### 3. Trait divergence is the result of in situ evolution

A putative character displacement pattern could be the result of ecological sorting as opposed to the in situ evolution of divergence in character. This alternative hypothesis can be rejected if the phenotypic range of a population in sympatry expands beyond what is found in any of the allopatric populations (Losos 2000; Schluter 2000a). Many cases of character displacement in plants show this pattern. For example, *Phlox drummondii* only has red corollas when in contact with *Phlox cuspidata* (Levin 1985), and *Opuntia echinocarpa* is shorter and narrower only when in contact with other cacti species (Cody 1991). This alternative hypothesis can also be rejected if trait means of a single species vary across communities in a pattern consistent with trait overdispersion (Schluter 2000a,b2000b). This pattern is found in seed mass in *Pinus* assemblages (Veech et al. 2000) and timing of pollen release in *Acacia* assemblages (Stone et al. 1998).

One method for testing such a pattern is to generate null models that distinguish between ecological sorting and character displacement. When testing for character displacement in assemblages of *Burmeistera* species, Muchhala and Potts (2007) developed a null model for trait overdispersion caused by character displacement. The model randomly sorted species that occur in more than one assemblage across sites to generate a null model of what trait dispersion would look like if the species in each assemblage had evolved exsertion lengths of reproductive parts at random. Additionally, a phylogeny-based null model of expected trait dispersion of an assemblage of species was recently developed in the animal literature (Davies et al. 2012). This method could be equally applicable in plant systems.

#### 4. A shift in the trait of interest is associated with a shift in resource acquisition or reproductive interactions

For character displacement to explain the difference in a trait between regions of sympatry and allopatry, the displaced trait must be linked to a shift in resource acquisition or reproductive interactions. Otherwise, the putative character displacement pattern could be explained by any number of other factors, including selection on traits that enhance the ability to compete for the same resources, rather than selection to rely upon a different set of resources (Aarssen 1983). By capturing pollinating bats, Muchhala (2008) demonstrated that the shift in exsertion length of reproductive parts of *Burmeistera* plants was associated with a shift in the location of pollen deposition on bat bodies. The body of a pollinating bat is a resource for plants, and this resource was divided in terms of the specific location of pollen placement. Similarly, by conducting pollinator observations, Whalen (1978) found that a shift from a large to small-flowered morph seen in numerous species of *Solanum* when sympatric with congeners corresponded with a shift in the size of visiting pollinators.

#### 5. Strength of interspecific competition or reproductive interactions is positively correlated with the degree of phenotypic similarity between species

While the fourth criterion simply links a shift in a trait with a shift in resource acquisition or reproductive interactions, the fifth criterion addresses whether this shift actually reduces competition. If character displacement is a response to competition for limited resources, then individuals with similar phenotypes should interact more strongly. One method for addressing this criterion is to conduct common garden experiments where species pairs compete for a limited resource. One treatment would involve competition between similar phenotypes, while another treatment would involve competition between less similar phenotypes. For example, Muchhala and Potts (2007) used flight cage experiments with wild-caught bats to demonstrate that heterospecific pollen deposition between *Burmeistera* species pairs decreases as difference in exsertion length of reproductive parts between species increases. In cases where the diverged character results in spatial segregation, such as an evolved preference for shallower soil or deeper shade, the common garden design must include additional treatments that mimic this spatial heterogeneity. As discussed in more detail in the following section, common garden experiments can directly investigate the process of character displacement if the more similar phenotypes used in the experiment are drawn from allopatric populations and the more divergent phenotypes are drawn from sympatric populations (Martin and Harding 1981).

#### 6. Sympatric and allopatric sites have similar resource availability

Differences in resource availability between sympatric and allopatric sites could explain the differences in phenotypes and reproductive compatibilities between sites. For example, reproductive isolation through flowering time and mating system differences between *Mimulus guttatus* and *Mimulus nasutus* in the western United States is likely a by-product of the local adaptation of *M. nasutus* in response to dry soil conditions, rather than a direct result of selection for reproductive isolation (Kiang and Hamrick 1978; Martin and Willis 2006).

It is nearly impossible to rule out the possibility that differences between sympatric and allopatric populations could be due to differences in resource availability between sites without experimentally manipulating the environment through reciprocal transplants or common garden designs. One exception may be large-scale comparative studies that separate adaptation in response to competitors from adaptation to local resources at a macro level by looking across many species pairs. A study of 41 sister-species pairs across three plant families in the Cape Floristic Region of South Africa found that shifts in pollination system follow adaptation to edaphic conditions only for sympatric sister species, suggesting that selection favors reproductive isolation in sympatry (van der Niet et al. 2006). If experimental designs or macro-level comparative studies are not possible, at a minimum, obvious differences in resources across sites, such as differences in water or light availability, differences in soil type, or differences in pollinator diversity or abundance, should be ruled out.

### Experimentally testing the process of character displacement

Over a decade ago, Schluter (2000a,b2000b) called for the direct experimental testing of character displacement hypotheses. He explained that if character displacement is the cause of an observed pattern, then experiments should test the process of character displacement by demonstrating that the intensity of competition declines in sympatric populations over time, and natural selection favors divergence in phenotypes among sympatric species. Testing these hypotheses offers the added benefit of also satisfying some or all of the six criteria for explaining observed patterns of character displacement. While Schluter has experimentally tested the process of character displacement in sticklebacks, few biologists in the animal or plant literature have followed his lead (Stuart and Losos 2013). The plant studies that have been conducted, however, demonstrate the experimental ease with which plants can be used in common gardens, selection analyses, and breeding designs to experimentally test character displacement hypotheses (Table 1). Plant biologists therefore have the opportunity to make a significant contribution to this new focus in character displacement research.

#### Testing whether the intensity of competition declines over time

If character displacement has occurred, the strength of competition between sympatric species should decline over time. One method for testing this hypothesis is to expose individuals from sympatric versus allopatric populations of a species to a competitor. If character displacement has occurred, then the intensity of competition should be greatest in the allopatric treatment. This competition experiment can be conducted in a greenhouse setting or in the field.

A field experiment was used to test whether *T. repens* had evolved in response to pairwise competition with multiple grass species (Turkington 1989). Ramets of *T. repens* were collected from a field from patches dominated by each of three different species of grasses. After the ramets were divided and grown in a greenhouse, some *T. repens* plants from each divided ramet were planted back into the field in competition with the competitor grass from their source site, and some were planted into competition with each of the other competitor grass species. The strength of competition was weakest when *T. repens* grew with the competitor from its source site, indicating that *T. repens* had evolved to reduce competition with its neighboring grass. While the design of this experiment is appropriate for studies of character displacement, this experiment falls outside of the character displacement literature because the specific trait that diverged to diminish competition was not identified.

In a study where the displaced trait was clear, potted plants of both color morphs of *Phlox drummondii* were placed into a natural population of *P. cuspidata,* and then the proportion of seed resulting from hybridization with *P. cuspidata* for each color morph was estimated (Levin 1985). The allopatric morph produced 38% hybrid seed while the sympatric morph produced only 13%, indicating that the sympatric morph had likely evolved to reduce competition for conspecific pollination. More recent work on this system used common garden experiments and pollinator observations to reveal that the sympatric morph is favored in the presence of *P. cuspidata* because it has an allele conferring dark pigmentation. This intense pigmentation encourages pollinator constancy by enabling pollinators to distinguish between the two species, thereby limiting heterospecific pollen transfer (Hopkins and Rausher 2012).

An interesting extension of these previous studies would involve identifying communities with varying ages of sympatry and examining how the intensity of competition changes over time. This design could reveal the rate of character displacement and whether this rate is consistent across populations with similar histories of sympatry.

#### Natural selection should favor divergence in phenotypes among sympatric species

Character displacement is the result of natural selection favoring a divergence in phenotypes between species competing for resources. Using classic statistical techniques, there are a number of experimental designs that can test whether the presence of a competitor alters natural selection on a focal plant species (Lande and Arnold 1983; Fishman and Wyatt 1999; Caruso 2000). For example, Caruso (2000) studied populations of *Ipomopsis aggregata* growing both with and without *Castilleja linariaefolia*, a competitor for hummingbird pollination. She measured selection on floral traits in both population types and found that the presence of the competitor resulted in increased selection on corolla length.

One issue with comparing selection in sites of sympatry versus allopatry is that resource availability may differ between these sites, and differences in selection may therefore be the result of differences in resources rather than the presence or absence of the competitor. Smith and Rausher (2008) addressed this issue when testing for selection for reproductive character displacement in *Ipomoea hederacea*. Rather than compare selection in sites of sympatry versus allopatry, they planted seeds of *I. hederacea* in a checkerboard pattern with its congener and competitor for pollinators *Ipomoea purpurea*. They then alternated between allowing pollinator visits to both species and preventing pollinator visits to the competitor species by covering inflorescences with bridal veil. They measured natural selection on *I. hederacea* with and without the presence of the competitor while keeping the resource base constant. They found that the presence of the competitor results in selection for an increase in the clustering of anthers about the stigma, which favors selfing over the risk of outcrossing with the wrong species.

If character displacement has progressed to the point where the displaced species are no longer competing, then it will not be possible to pick up a signal of altered natural selection (Connell 1980). This issue can be dealt with in a number of ways in plants. First, if there are allopatric populations available, then the study should be conducted by comparing selection on the allopatric individuals with and without the competitor. Second, if no allopatric population is available, then breeding designs can be arranged to select for a range of phenotypes that overlaps more with the competitor phenotype, and this wider range of phenotypes can be used for the selection experiment. Finally, a recent study on character displacement in *Phlox drummondii* demonstrated that population genetic analyses can be used to uncover the signature of a past selective sweep in sympatric populations (Hopkins et al. 2012).

## What are Some Additional Approaches for Studying Character Displacement in Plants?

Plant systems have proven to be especially amenable to the experimental designs required for testing character displacement hypotheses. In the following section, I explore some additional approaches that may be useful for uncovering examples of character displacement in plants.

### Island systems

Some of the most well-supported examples of character displacement in the animal literature come from island systems such as the studies of Darwin's finches in the Galapagos (Grant and Grant 2006) and *Anolis* lizards in the Caribbean (Losos 2009). Islands offer the opportunity to test the repeatability of character displacement, as there may be many occurrences of sympatry and allopatry across an island chain. Island chains are equally suitable to the study of character displacement in plants (Cody 1984; Miyake and Inoue 2003). Furthermore, plants are sessile organisms often with highly structured populations. Even on the mainland, then, limited gene flow between plant populations may result in island-like patterns of species distributions ideal for the study of character displacement.

### Species invasions

Species invasions offer the opportunity to capture the process of character displacement in action. When the time since the introduction of an invasive competitor is known across multiple communities, researchers can test for character displacement by investigating whether the strength of competition between a native and an invasive competitor declines as the time since invasion increases (Lankau et al. 2009). A greenhouse study using soil from source communities of varying stages of garlic mustard (*Alliaria petiolata*) invasion showed that populations of *Pilea pumila*, a native annual from these same source communities, had adapted to the specific soil qualities present at each invasion stage (Lankau 2013). While the exact displaced character that permits coexistence is not clear, a previous study suggests that *P. pumila* populations that are experienced with *A. petiolata* have evolved to maintain their beneficial arbuscular mycorrhizal fungi connections even in the presence of allelochemicals released by the invader (Lankau 2012).

There are currently many examples of ecologically similar native and invasive species in direct competition with one another. The invasive jewelweed *Impatiens glandulifera* competes with the native congener *Impatiens capensis* in communities throughout New York, Massachusetts, and Maine (Tabak and von Wettberg 2008). Native and introduced subspecies of *Phragmites* are commonly found competing in the eastern United States (Meyerson et al. 2010). Native and invasive populations of the dandelion, *Taraxacum*, are found competing for pollinators throughout Japan (Kandori et al. 2009). All of these examples offer promising systems for the study of character displacement in plants.

### A closer look

Perhaps the greatest challenge to the study of character displacement in plants is identifying which character traits are likely involved in competition for resources and therefore likely to be displaced. Studies of reproductive character displacement have successfully recognized shifts in visible traits such as flower color and style length. More cryptic reproductive traits such as floral scent, sugar and amino acid concentrations in nectar, pollen to ovule ratios, and subtle alterations in floral and inflorescence structure should also be investigated, as these traits may be equally susceptible to character displacement (Lindsey and Bell 1985).

Studies of ecological character displacement can also benefit from a closer look at the subtle traits involved in resource acquisition. Character displacement patterns have been noticed in clearly visible morphological traits such as growth form and seed mass, but more subtle morphological traits such as rooting structure and depth, specific leaf area, and petiole length may also be under selection for character divergence (Cody 1991; Veech et al. 2000). Furthermore, physiological traits such as the specific forms, ratios, and timing of nutrients absorbed should be explored (McKane et al. 1990; Ashton et al. 2010). Finally, a deeper investigation of developmental traits such as growth rate and life history may also provide examples of ecological character displacement in plants (Tanaka et al. 2008). Even once a diverged trait is identified, proving that it is solely responsible for the observed niche shift is challenging. The experimental designs presented here, however, should reveal whether the trait is at least partially responsible.

## Conclusion

The existing studies of character displacement in plants do not yet provide irrefutable evidence that character displacement is a common response to interspecific competitive and reproductive interactions. They do, however, suggest that character displacement plays an important role in minimizing competition in at least some plant communities (Table 1). It is time to use these studies as a springboard to continue with rigorous testing of character displacement hypotheses in plants. The experimental designs for testing character displacement hypotheses are now readily available, and plant systems are especially amenable to these designs because of the experimental ease with which they can be used in common gardens, selection analyses, and breeding designs. Using these experimental approaches to test for ecological character displacement is especially critical as this area of research is mostly unexplored. By focusing greater attention on character displacement in plants, we have the potential to enhance our fundamental understanding of the ecological and evolutionary forces that shape plant communities.

## Supporting Information

Table S1 List of animal and plant studies published from 1956 thorough December of 2013 with “character displacement” in the title. These studies were included in Figure 1.

Table S2 List of additional plant character displacement studies published from 1956 through December 2013 that use an alternate term to describe character displacement in the title. These studies were included in Figure 1.
